# Analytical framework and data for evaluating a City Resilience Strategy’s emphasis on social equity and justice

**DOI:** 10.1016/j.dib.2019.104328

**Published:** 2019-08-27

**Authors:** Joanne Fitzgibbons, Carrie L. Mitchell

**Affiliations:** School of Planning, University of Waterloo, Canada

**Keywords:** Justice, Resilience, Content analysis, Plan evaluation

## Abstract

This article contains four data tables: 1 and 2: A content analysis framework for evaluating the degree to which urban resilience plans emphasize issues of justice and equity in plan content, and associated point rubric for scoring criteria; 3. The raw numerical data collected for a plan evaluation where we deployed this framework to analyze a sample of 31 strategies from the “100 Resilient Cities – Pioneered by the Rockefeller Foundation” (100RC) initiative; and, 4. Inter-rater reliability scores for this plan evaluation.

This dataset accompanies a 2019 article submitted to the journal World Development titled: *Just urban futures? Exploring equity in “100 Resilient Cities”.*

**Table:** Specifications Table

Subject area	Planning; International Development;
More specific subject area	Resilience; Equity planning
Type of data	Tables
How data was acquired	The data was acquired through content analysis of 31 City Resilience Strategies released under “100 Resilient Cities – Pioneered by the Rockefeller Foundation” (100RC).
Data format	Analytical framework, and concise numerical data.
Experimental factors	An analytical framework was developed and then used to conduct a content analysis of 31 published City Resilience Strategies released under 100RC. However, our purposive sample deliberately excluded several strategies from the United States of America (USA) in order to capture a more proportionate sample across Global North and South countries.
Experimental features	The attached analytical framework was developed based on a review of relevant resilience and justice literature. We used the framework to extract quantitative observations from strategy content about the degree to which they prioritized social equity. For some criteria, the framework also guided the collection of qualitative observations.
Data source location	We reviewed the content of 31 plans from around the globe in the following cities: Amman, Jordan Athens, Greece Bangkok, Thailand Bristol, UK Byblos, Lebanon Cali, Colombia Greater Christchurch, New Zealand Da Nang, Vietnam Dakar, Senegal Dallas, USA Glasgow, UK Medellin, Colombia Melbourne, Australia Mexico City, Mexico Montreal, Canada Norfolk, USA Paris, France Quito, Ecuador Ramallah, Palestine Rio de Janeiro, Brazil Rome, Italy Rotterdam, The Netherlands San Francisco, USA Santa Fe, Argentina Santiago de Chile, Chile Semarang, Indonesia Surat, India Thessaloniki, Greece Toyama, Japan Vejle, Denmark Wellington, New Zealand
Data accessibility	The data is with this article.
Related research article	This data is associated with a submission to the Elsevier journal “World Development”: Fitzgibbons, J. and C. Mitchell (in press). Just urban futures? Exploring equity in “100 Resilient Cities”. *World Development.*

**Table:** 

**Value of the Data** • The results of our analysis can be triangulated with existing and future empirical work exploring justice and resilience, for example, by: providing a global analysis to complement more locally-based case studies; providing data to compare instances of resilience planning against plans created under a different framing, eg. sustainability; and, comparing to later iterations of resilience planning either in later 100RC cohorts or from different initiatives. • Planners are increasingly recognizing the importance of equity and justice to their practice, but these normative issues can be challenging to monitor and quantify. Our framework can be used by researchers to analyze the extent to which these issues are reflected in plan content, and practitioners can use our framework as a template for incorporating equity into the design of urban resilience (and other) plans. • With minor adjustments (such as the addition of new issue-specific criteria), our framework can be used beyond urban resilience planning to support the evaluation and integration of equity in other urban plans, such as (but not limited to) housing strategies, or economic development strategies.

## 1 Data

This Data in Brief submission contains an analytical framework for evaluating the degree to which urban plans prioritize social equity in both process and outcomes, as well as the numerical results of a plan evaluation where the authors deployed this framework to analyze 31 City Resilience Strategies produced under “100 Resilient Cities – Pioneered by the Rockefeller Foundation”.

This article contains a series of tables:

1. The first table, titled “Point rubric” is used to interpret and assign points in the Evaluation Framework.
2. The second table, “Evaluation Framework”, is the framework and list of indicators that we used to score the strategies based on strategy content. This table is needed to interpret the values conveyed in the “Results by question and city” table. Each criteria is numbered in Column A, which corresponds directly with Row 2 in the “Results by question and city” table. For example: The city Amman, Jordan received a score of 1 on Question 1, a score on 0.5 on Question 2, etc.
3. The third table, “Results by criteria and city”, express how each of the 31 Cities in our study scored on each of the criteria expressed in the “Analytical Criteria”.
4. The fourth table, “Inter-rate reliability”, expresses the differences in ratings between the first author (who conducted the analysis) and the second rater (who independently repeated the analysis “blind” [without having seen first author's results]) This process was conducted to mitigate the likelihood of reviewer positionalities biasing the analysis, given the normative nature of the study.

## 2 Experimental design, materials, and methods

### 2.1 Analytical framework creation and evaluation technique

The analytical framework included in this Data in Brief article was developed by the authors following a review of relevant literature exploring urban resilience, justice, and social equity in planning processes [1], [2], [3], [4], [5], [6], [7], [8], [9], [10], [11], [12], [13], [14], [15].

The authors distilled this literature into a set of 36 criteria, of which 28 can be used to assign scores. The remaining eight unscored criteria are used to guide the collection of qualitative observations.

Each scoreable criteria can be assigned either a full point (1), a partial point (0.5), or no points (0) depending on how thoroughly the strategy addresses the criteria. For example, a strategy that discusses a particular criteria in great detail may receive a score of 1; a strategy that only partially or superficially mentions the criteria might receive a score of 0.5; and, a strategy that does not acknowledge the issue mentioned in the criteria would receive a 0.

### 2.2 Sampling strategy

Because the accompanying research article (Fitzgibbons & Mitchell, 2019) sought to take a comparative development lens, exploring the implementation of 100RC across developed and less developed countries, our purposive sample deliberately excluded some strategies from the United States of America (USA). This was done because wealthier countries – particularly the USA – make up a large proportion of participating cities in 100RC, and the inclusion of all published USA strategies might have biased the results of our analysis toward the experience of more developed countries and the USA in particular. Accordingly, only 3 published strategies from the USA were selected, one each from Western, Eastern and Southern regions of the country.

**Fig. 1:**
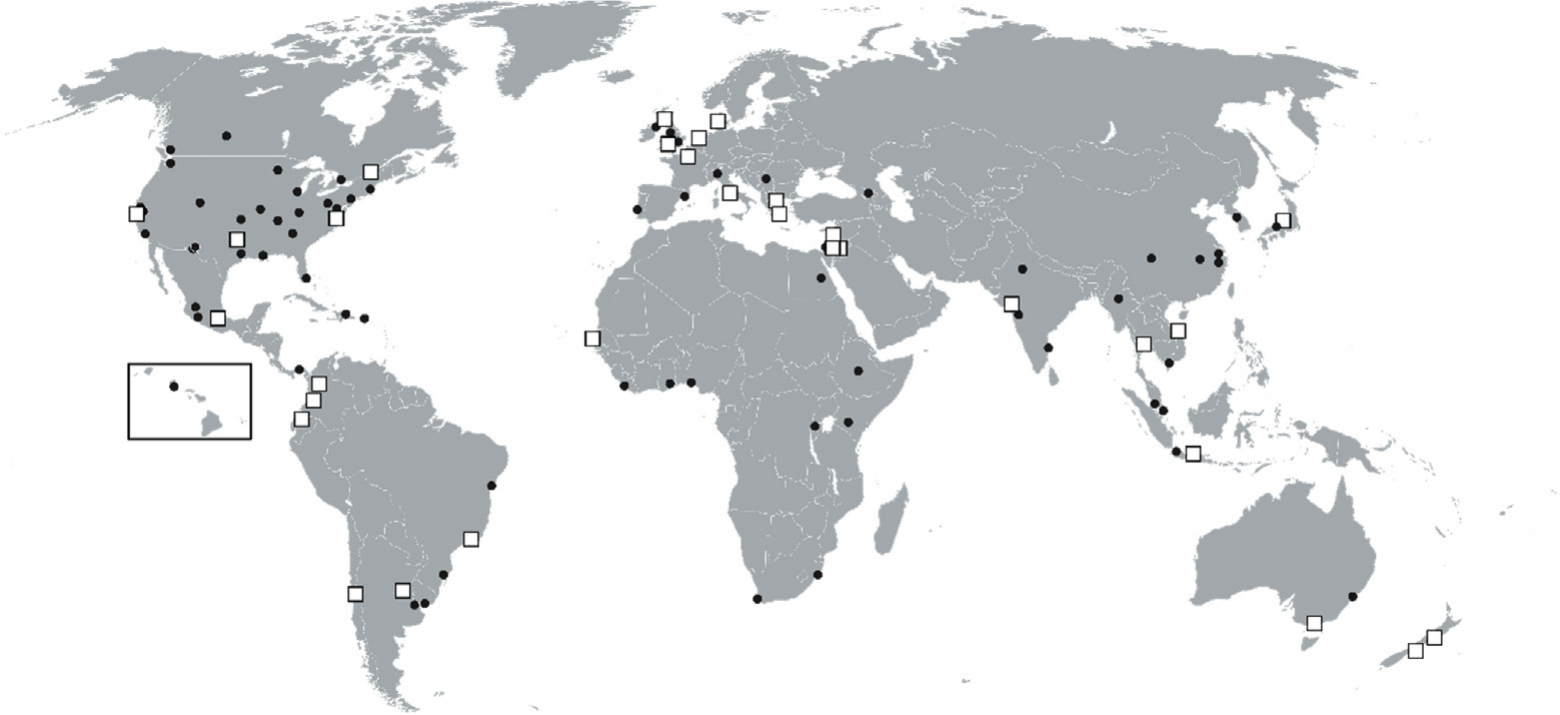
Sampled cities (squares) and all participating 100 Resilient Cities (dots). Source: Fitzgibbons & Mitchell, 2019 [16].

Apart from the aforementioned exclusion criteria, all 100RC City Resilience Strategies that were published in English as of September, 2018 underwent content analysis using the analytical framework in this article. Fig. 1 contextualizes our sample within the broader 100RC network of member cities.

### 2.3 Inter-rater reliability

To reduce the potential for bias, the content analysis was conducted by the first author and also by a second rater who had no prior knowledge of the study's hypothesis or findings thus far.

The first author and second rater met to review discrepancies and repeated the analysis a third time, together, on any strategy with major (more than 20% different) and minor (between 10% and 20% different) discrepancies in the final score. The scores for 10 City Resilience Strategies were changed during this reconciliation.

The similarity between raters was 97.35% before meeting to reconcile differences. After this meeting, the reconciled results were 99.31% similar.

## Acknowledgments

This work was supported by the Social Sciences and Humanities Research Council of Canada (SSHRC) Insight Development Grants (#430-2017-00135) and Canada Graduate Scholarships program, and the Province of Ontario's Ontario Graduate Scholarship. We would also like to thank Kristen Rieger for her inter-rater reliability support.

## Conflict of interest

The authors declare that they have no known competing financial interests or personal relationships that could have appeared to influence the work reported in this paper.
