# Supplementary material for: Analytical framework and data for evaluating a City Resilience Strategy’s emphasis on social equity and justice
Source: Data Brief. 2019 Aug 27;26:104328. doi: 10.1016/j.dib.2019.104328 (PMC6743002; doi:10.1016/j.dib.2019.104328)
Supplement: Multimedia component 1 [file mmc1.docx]

**Explanation to interpret the tables herein**

The first table (1), “**Point Rubric**”, is a point rubric to interpret or assign scored to coded content from the strategy or plan. It explains how points were allocated depending on how thoroughly the criteria were fulfilled.

The second table (2), “**Evaluation Framework**”, is the evaluation framework and list of indicators that were used to score strategies based on their written content. This sheet is needed to interpret the values conveyed in the third table (3), “Results by criteria and city”. Each criteria is numbered in the first column of Table 2. This column corresponds directly with Row 2 of “Results by criteria and city”.
*For example: The city Amman, Jordan received a score of 1 on Question 1 (it fulfilled the criteria), a score of 0.5 on Question 2 (it partially fulfilled the criteria), and so on.*

The third table (3), “**Results by criteria and city**”, includes the numerical data expressing how each city strategy scored in relation to the criteria from Table 2.

The fourth table (4), “**Inter-rater reliability**”, expresses the differences in ratings between the first author (who conducted the content analysis) and a second independent rater (who repeated the analysis “blind”, without having seen the first author’s results).
The author and second rater met to review major discrepancies and repeated the analysis together for a third time on strategies that had major discrepancies in ratings. The scores for 10 City Resilience Strategies were changed during this reconciliation.
The similarity of scores between raters was 97.35% similar before reconciling differences. After reconciling, the results were 99.31% similar.

1. **Point rubric**

| **Meaning** | **Points assigned** |
| --- | --- |
| There is evidence of proactive effort on these grounds. The criteria was clearly fulfilled. | 1 |
| There is only superficial mention of this criteria, or the answer is unclear, or the criteria is only partly fulfilled. | 0.5 |
| The possibility of this issue is not acknowledged or discussed. The criteria is not fulfilled at all. | 0 |

1. **Evaluation Framework**

The numbers in Column A of this sheet are the "criteria number/code", and correspond with Row 2 in the "Results by question and city" tab.

| **Recognitional and Distributive Justice** | |  |
| --- | --- | --- |
|  | Acts of Omission |  |
| 1 | Are specific populations of interest *(eg. vulnerable groups)* identified in the strategy? | 1 |
| 2 | - If yes, is their vulnerability explored in-depth? *(eg. historic or structural reasons for their vulnerability described)* | 1 |
| 3 | Are benefits intentionally directed at specific groups? | 1 |
| 4 | - Why or why not? (unscored) | - |
| 5 | Are there some benefits that may not be accessible to vulnerable stakeholders? | - |
| 6 | - Why or why not? (unscored) | - |
| 7 | - Is this acknowledged in the Strategy? | 1 |
| 8 | - Is there evidence that the Strategy attempts to mitigate this or improve access? | 1 |
| 9 | Is the strategy available in the predominant local language and/or minority languages? (unscored) | - |
|  | *TOTAL POSSIBLE POINTS* | *5* |
|  | Acts of Commission |  |
| 10 | Could any of the proposed actions in the strategy directly or indirectly negatively affect a vulnerable group? *(eg. through displacement)* | - |
| 11 | - Does the strategy acknowledge this potential impact? | 1 |
| 12 | - Do any proposed actions attempt to mitigate this impact? | 1 |
| 13 | Does the strategy propose any actions to correct previous acts of commission or injustice? | 1 |
|  | *TOTAL POSSIBLE POINTS* | *3* |
|  | Vulnerability |  |
| 14 | Is vulnerability explicitly defined and explained? | 1 |
| 15 | Does the strategy’s understanding of vulnerability include socioeconomic/sociocultural characteristics? *(eg. more than just risk exposure)* | 1 |
| 16 | Does the strategy feature a map that describes socioeconomic vulnerability or human development? | 1 |
| 17 | Does the strategy acknowledge how municipal systems or processes might exacerbate vulnerabilities? | 1 |
| 18 | Who is assigned responsibility or accountability for uneven vulnerability? | - |
| 19 | Are the root causes of vulnerability addressed in the actions, or do actions attempt to “treat” rather than prevent vulnerability? | - |
|  | *TOTAL POSSIBLE POINTS* | *4* |
| **Procedural Justice** | |  |
|  | Monitoring and Evaluation |  |
| 20 | Does the strategy describe a framework for evaluating whether or not its actions have been successful? | 1 |
| 21 | Does it mention that equity considerations will feature as an indicator of success/failure? | 1 |
| 22 | Does the strategy mention that monitoring and evaluation protocol will be collaboratively designed? | 1 |
| 23 | Is there an opportunity for public participation in conducting monitoring and evaluation? | 1 |
|  | *TOTAL POSSIBLE POINTS* | *4* |
|  | Transparency and Participation |  |
| 24 | Is the stakeholder engagement process described? | 1 |
| 25 | - Does the strategy describe during which phases participation took place? | 1 |
| 26 | - Does the strategy describe what media or techniques were used for consultation? | 1 |
| 27 | - Does the strategy describe how many people were consulted? | 1 |
| 28 | - Does the strategy describe engaging external stakeholders in early problem definition? | 1 |
| 29 | - Does the strategy describe engaging external stakeholders in co-creation and solution generation? | 1 |
| 30 | - Does the strategy describe how information was disseminated to the non-participant public? *(eg. general public communications)* | 1 |
| 31 | Which stakeholders are identified and targeted for consultation? | - |
| 32 | - Is there evidence that vulnerable groups were afforded an opportunity to self-identify their needs and priorities? | 1 |
| 33 | - Is there mention of vulnerable residents being engaged as stakeholders or participants? | 1 |
| 34 | - Were specific partnerships or arrangements achieved with key external stakeholders? *(eg. civil society, associations, industry)* | 1 |
| 35 | Does the strategy describe what rationale was used to identify and recruit stakeholders? *(eg. why some actors were included while others were not)* | 1 |
| 36 | Are there plans for ongoing participation, or is the strategy portrayed as “finished”? | 1 |
|  | *TOTAL POSSIBLE POINTS* | *12* |
|  |  |  |
|  | *GRAND TOTAL POSSIBLE POINTS* | *28* |

1. **Results by criteria and city**

|  | Recognitional and Distributive Justice | | | | | | | | | | | | | | | | | | | Procedural Justice | | | | | | | | | | | | | | | | |  |
| --- | --- | --- | --- | --- | --- | --- | --- | --- | --- | --- | --- | --- | --- | --- | --- | --- | --- | --- | --- | --- | --- | --- | --- | --- | --- | --- | --- | --- | --- | --- | --- | --- | --- | --- | --- | --- | --- |
|  | 1 | 2 | 3 | 4 | 5 | 6 | 7 | 8 | 9 | 10 | 11 | 12 | 13 | 14 | 15 | 16 | 17 | 18 | 19 | 20 | 21 | 22 | 23 | 24 | 25 | 26 | 27 | 28 | 29 | 30 | 31 | 32 | 33 | 34 | 35 | 36 | **Total** |
| Amman | 1 | 0.5 | 1 | - | - | - | 0 | 0 | - | - | 0 | 0 | 0 | 0 | 0.5 | 0 | 0 | - | - | 1 | 0.5 | 0 | 0 | 1 | 1 | 1 | 0 | 1 | 1 | 0 | - | 0 | 1 | 1 | 0 | 1 | **12.5** |
| Athens | 1 | 1 | 1 | - | - | - | 0 | 0 | - | - | 0 | 0 | 0 | 0 | 1 | 1 | 0 | - | - | 1 | 1 | 0 | 0 | 1 | 1 | 1 | 1 | 1 | 1 | 0 | - | 1 | 1 | 1 | 0 | 1 | **17** |
| Bangkok | 1 | 0.5 | 1 | - | - | - | 1 | 0 | - | - | 0 | 0 | 0 | 0 | 1 | 0 | 0.5 | - | - | 0 | 0 | 0 | 0 | 1 | 1 | 0 | 1 | 1 | 1 | 0 | - | 0 | 0 | 1 | 0 | 0 | **11** |
| Bristol | 1 | 0 | 1 | - | - | - | 1 | 1 | - | - | 0.5 | 0.5 | 0 | 0 | 0.5 | 0 | 0.5 | - | - | 0.5 | 0.5 | 0 | 0 | 1 | 0 | 1 | 1 | 1 | 1 | 1 | - | 0 | 0.5 | 1 | 1 | 1 | **16.5** |
| Byblos | 0.5 | 0 | 0.5 | - | - | - | 0 | 0 | - | - | 0 | 0 | 0 | 0 | 1 | 0.5 | 0 | - | - | 1 | 0 | 0 | 0.5 | 0.5 | 1 | 1 | 1 | 1 | 1 | 0 | - | 0 | 0.5 | 0 | 0 | 1 | **11** |
| Cali | 1 | 0 | 1 | - | - | - | 0 | 0 | - | - | 0 | 1 | 0 | 0 | 0.5 | 0 | 0 | - | - | 1 | 0.5 | 0 | 0 | 1 | 1 | 1 | 1 | 1 | 0.5 | 0 | - | 1 | 1 | 1 | 0 | 0 | **13.5** |
| Christchurch | 1 | 1 | 1 | - | - | - | 0.5 | 0 | - | - | 0 | 0.5 | 1 | 0.5 | 1 | 1 | 0.5 | - | - | 0 | 0 | 0.5 | 1 | 1 | 1 | 1 | 1 | 1 | 1 | 0 | - | 1 | 0 | 1 | 0 | 1 | **18.5** |
| Da Nang | 1 | 0.5 | 1 | - | - | - | 0 | 0 | - | - | 1 | 0 | 0 | 0 | 1 | 0 | 0 | - | - | 1 | 0 | 0 | 0.5 | 1 | 1 | 1 | 0 | 0.5 | 0 | 0 | - | 0 | 0 | 0 | 0 | 1 | **10.5** |
| Dakar | 0 | 0 | 0.5 | - | - | - | 0 | 0 | - | - | 0 | 0 | 0 | 0 | 0 | 0 | 0 | - | - | 0.5 | 0 | 0 | 0 | 1 | 1 | 1 | 0 | 1 | 1 | 0.5 | - | 0.5 | 0 | 1 | 0 | 0.5 | **8.5** |
| Dallas | 1 | 1 | 1 | - | - | - | 0 | 1 | - | - | 0 | 0.5 | 1 | 0 | 1 | 1 | 1 | - | - | 1 | 1 | 1 | 0 | 1 | 1 | 1 | 1 | 1 | 1 | 0 | - | 0 | 0 | 1 | 0 | 0 | **18.5** |
| Glasgow | 1 | 1 | 1 | - | - | - | 1 | 1 | - | - | 0 | 0.5 | 0 | 0 | 1 | 0 | 0 | - | - | 1 | 1 | 0.5 | 0.5 | 1 | 1 | 1 | 1 | 1 | 1 | 1 | - | 1 | 1 | 1 | 1 | 1 | **21.5** |
| Medellin | 1 | 0 | 1 | - | - | - | 0 | 0 | - | - | 0 | 0.5 | 1 | 0 | 1 | 0 | 0 | - | - | 0.5 | 0.5 | 0 | 0 | 0 | 0 | 0 | 0 | 1 | 1 | 0.5 | - | 0.5 | 0.5 | 1 | 0 | 1 | **11** |
| Melbourne | 1 | 1 | 0.5 | - | - | - | 0 | 0 | - | - | 0 | 0 | 0 | 0.5 | 1 | 1 | 0.5 | - | - | 1 | 0 | 1 | 0 | 1 | 1 | 1 | 1 | 1 | 1 | 0 | - | 0 | 0 | 1 | 0.5 | 1 | **16** |
| Mexico City | 1 | 1 | 1 | - | - | - | 1 | 1 | - | - | 0 | 0 | 1 | 1 | 1 | 1 | 1 | - | - | 1 | 0 | 0 | 0.5 | 1 | 1 | 1 | 0 | 1 | 1 | 0 | - | 0 | 0 | 1 | 0 | 1 | **18.5** |
| Montreal | 0.5 | 0.5 | 0 | - | - | - | 0 | 0 | - | - | 0 | 0 | 0 | 1 | 1 | 0.5 | 0 | - | - | 1 | 0 | 0 | 0 | 1 | 1 | 1 | 1 | 1 | 1 | 0 | - | 0 | 0 | 1 | 0 | 1 | **12.5** |
| Norfolk | 0 | 0 | 1 | - | - | - | 0 | 0 | - | - | 0 | 0 | 0 | 0 | 1 | 0.5 | 1 | - | - | 0.5 | 0 | 0 | 0 | 0.5 | 0 | 0.5 | 0.5 | 1 | 0 | 0 | - | 0 | 0 | 1 | 0 | 1 | **8.5** |
| Paris | 1 | 1 | 0 | - | - | - | 0 | 0 | - | - | 0 | 0 | 0 | 0 | 1 | 0 | 0.5 | - | - | 0 | 0 | 0 | 0 | 1 | 1 | 1 | 0.5 | 1 | 1 | 0 | - | 0.5 | 0 | 1 | 0 | 0 | **10.5** |
| Quito | 0.5 | 0 | 1 | - | - | - | 0 | 0 | - | - | 1 | 0.5 | 0 | 0 | 0.5 | 1 | 0 | - | - | 1 | 0 | 0 | 0 | 1 | 1 | 1 | 0 | 1 | 1 | 0 | - | 0 | 0.5 | 1 | 0 | 1 | **13** |
| Ramallah | 1 | 1 | 0.5 | - | - | - | 0 | 0 | - | - | 0 | 0 | 0.5 | 1 | 1 | 0.5 | 0 | - | - | 1 | 0 | 0 | 0 | 1 | 1 | 1 | 1 | 1 | 1 | 0.5 | - | 0 | 0.5 | 1 | 0 | 1 | **15.5** |
| Rio de Janeiro | 1 | 0.5 | 1 | - | - | - | 0 | 0 | - | - | 0 | 0 | 0 | 0 | 1 | 1 | 1 | - | - | 1 | 1 | 0 | 0 | 1 | 1 | 1 | 0.5 | 0 | 1 | 0 | - | 0.5 | 0 | 0 | 0 | 0.5 | **13** |
| Rome | 0.5 | 0 | 1 | - | - | - | 1 | 0 | - | - | 0 | 0 | 0 | 0 | 1 | 1 | 1 | - | - | 1 | 0 | 0 | 0 | 1 | 1 | 1 | 1 | 1 | 0 | 0 | - | 0.5 | 0 | 0.5 | 1 | 1 | **14.5** |
| Rotterdam | 1 | 0 | 0.5 | - | - | - | 0 | 0 | - | - | 0 | 0 | 0 | 0 | 0.5 | 0 | 0 | - | - | 0 | 0 | 0 | 0 | 0 | 0 | 0 | 0.5 | 0.5 | 0 | 0 | - | 0 | 0 | 1 | 0 | 1 | **5** |
| San Francisco | 1 | 0 | 1 | - | - | - | 0 | 0 | - | - | 0 | 0 | 0 | 0 | 0.5 | 0 | 0 | - | - | 1 | 0 | 1 | 0 | 1 | 0 | 0.5 | 0.5 | 1 | 0.5 | 0 | - | 0 | 0 | 0 | 0 | 1 | **9** |
| Santa Fe | 1 | 0.5 | 1 | - | - | - | 0 | 0 | - | - | 1 | 1 | 0 | 0 | 1 | 1 | 0.5 | - | - | 0 | 0 | 0 | 0 | 1 | 1 | 1 | 1 | 1 | 1 | 0 | - | 0 | 0.5 | 1 | 0 | 1 | **15.5** |
| Santiago de Chile | 1 | 1 | 1 | - | - | - | 0 | 0 | - | - | 0 | 0 | 1 | 0 | 1 | 1 | 1 | - | - | 0 | 0 | 0 | 0 | 1 | 1 | 1 | 1 | 1 | 1 | 0 | - | 0.5 | 0 | 1 | 0 | 1 | **15.5** |
| Semarang | 1 | 0 | 1 | - | - | - | 0 | 0 | - | - | 0 | 0 | 0 | 0 | 1 | 0 | 0 | - | - | 1 | 0 | 0 | 0 | 1 | 1 | 1 | 0 | 1 | 1 | 0 | - | 0 | 0 | 1 | 0 | 1 | **11** |
| Surat | 1 | 0.5 | 0 | - | - | - | 0 | 0 | - | - | 0 | 0 | 0 | 0 | 0 | 0 | 0 | - | - | 0 | 0 | 0 | 0 | 1 | 0 | 1 | 0 | 1 | 1 | 0.5 | - | 0 | 0 | 1 | 0.5 | 1 | **8.5** |
| Thessaloniki | 1 | 0.5 | 1 | - | - | - | 0 | 0 | - | - | 0 | 0.5 | 0 | 0 | 1 | 0 | 0 | - | - | 1 | 0 | 0 | 0 | 1 | 1 | 1 | 1 | 1 | 1 | 0 | - | 0 | 0 | 1 | 0 | 0.5 | **12.5** |
| Toyama | 1 | 0.5 | 1 | - | - | - | 0 | 0 | - | - | 0 | 0 | 0 | 0 | 0 | 0 | 0 | - | - | 1 | 0 | 0 | 0 | 1 | 0 | 0 | 0 | 1 | 1 | 0 | - | 0 | 0 | 1 | 0 | 1 | **8.5** |
| Vejle | 1 | 0 | 1 | - | - | - | 0.5 | 0.5 | - | - | 0 | 0 | 0 | 0 | 1 | 0 | 0 | - | - | 0.5 | 0 | 0 | 0 | 1 | 1 | 1 | 0 | 1 | 1 | 0 | - | 0 | 0 | 1 | 1 | 1 | **12.5** |
| Wellington | 1 | 0 | 1 | - | - | - | 0 | 0 | - | - | 0 | 0 | 0 | 0 | 1 | 1 | 0 | - | - | 0.5 | 0 | 0 | 0 | 1 | 1 | 1 | 1 | 1 | 1 | 1 | - | 0 | 0 | 1 | 0 | 1 | **13.5** |
| TOTALS | 28 | 15 | 27 | - | - | - | 7 | 5.5 | - | - | 4 | 6 | 6.5 | 5 | 26 | 14 | 10 | - | - | 21 | 6.5 | 4.5 | 3.5 | 29 | 25 | 27 | 20 | 30 | 27 | 6 | - | 8 | 8 | 28 | 6 | 27 |  |
| % of S* that obtained | 90 | 47 | 85 | - | - | - | 23 | 18 | - | - | 13 | 19 | 21 | 16 | 84 | 45 | 32 | - | - | 68 | 21 | 15 | 11 | 94 | 81 | 87 | 63 | 97 | 87 | 19 | - | 26 | 26 | 89 | 19 | 85 |  |

1. **Inter-rater reliability scores**

|  | **Rater's o. score** | **As percentage** | **Author's o. score** | **As percentage** | **Difference (pts)** | **Difference (%)** | **Altered score** | **After reconciling** |
| --- | --- | --- | --- | --- | --- | --- | --- | --- |
| Amman | 14.5 | 51.79% | 12.5 | 44.64% | 2 | 7.14% |  | 7.14% |
| Athens | 19 | 67.86% | 17 | 60.71% | 2 | 7.14% |  | 7.14% |
| Bangkok | 12.5 | 44.64% | 11 | 39.29% | 1.5 | 5.36% |  | 5.36% |
| Bristol | 8.5 | 30.36% | 16.5 | 58.93% | -8 | -28.57% | 17 | 0.00% |
| Byblos | 11.5 | 41.07% | 11 | 39.29% | 0.5 | 1.79% |  | 1.79% |
| Cali | 17 | 60.71% | 13.5 | 48.21% | 3.5 | 12.50% | 14 | 0.00% |
| Da Nang | 11 | 39.29% | 10.5 | 37.50% | 0.5 | 1.79% |  | 1.79% |
| Dakar | 11 | 39.29% | 8.5 | 30.36% | 2.5 | 8.93% |  | 8.93% |
| Dallas | 19 | 67.86% | 18.5 | 66.07% | 0.5 | 1.79% |  | 1.79% |
| Glasgow | 13.5 | 48.21% | 21.5 | 76.79% | -8 | -28.57% | 22 | 0.00% |
| Christchurch | 12.5 | 44.64% | 18.5 | 66.07% | -6 | -21.43% | 19 | 0.00% |
| Medellin | 9 | 32.14% | 11 | 39.29% | -2 | -7.14% |  | -7.14% |
| Melbourne | 13.5 | 48.21% | 16 | 57.14% | -2.5 | -8.93% |  | -8.93% |
| Mexico City | 19 | 67.86% | 18.5 | 66.07% | 0.5 | 1.79% |  | 1.79% |
| Montreal | 11 | 39.29% | 12.5 | 44.64% | -1.5 | -5.36% |  | -5.36% |
| Norfolk | 10.5 | 37.50% | 8.5 | 30.36% | 2 | 7.14% |  | 7.14% |
| Paris | 11 | 39.29% | 10.5 | 37.50% | 0.5 | 1.79% |  | 1.79% |
| Quito | 19.5 | 69.64% | 13 | 46.43% | 6.5 | 23.21% | 13 | 0.00% |
| Ramallah | 14 | 50.00% | 15.5 | 55.36% | -1.5 | -5.36% |  | -5.36% |
| Rio | 9.5 | 33.93% | 13 | 46.43% | -3.5 | -12.50% | 13 | 0.00% |
| Rome | 12.5 | 44.64% | 14.5 | 51.79% | -2 | -7.14% |  | -7.14% |
| Rotterdam | 7 | 25.00% | 5 | 17.86% | 2 | 7.14% |  | 7.14% |
| San Francisco | 9 | 32.14% | 9 | 32.14% | 0 | 0.00% |  | 0.00% |
| Santa Fe | 12.5 | 44.64% | 15.5 | 55.36% | -3 | -10.71% | 16 | 0.00% |
| Santiago de Chile | 15 | 53.57% | 15.5 | 55.36% | -0.5 | -1.79% |  | -1.79% |
| Semarang | 9 | 32.14% | 11 | 39.29% | -2 | -7.14% |  | -7.14% |
| Surat | 11 | 39.29% | 8.5 | 30.36% | 2.5 | 8.93% |  | 8.93% |
| Thessaloniki | 9.5 | 33.93% | 12.5 | 44.64% | -3 | -10.71% | 13 | 0.00% |
| Toyama | 4.5 | 16.07% | 8.5 | 30.36% | -4 | -14.29% | 8.5 | 0.00% |
| Vejle | 13.5 | 48.21% | 12.5 | 44.64% | 1 | 3.57% |  | 3.57% |
| Wellington | 10 | 35.71% | 13.5 | 48.21% | -3.5 | -12.50% | 14 | 0.00% |
| SUM / AVG | 380.5 | 43.84% | 403.5 | 46.49% | -2.65% | -2.65% |  | 0.69% |
| PERCENT SIMILAR |  |  |  |  |  | 97.35% |  | 99.31% |
